# Differentiation of claustrum resting‐state functional connectivity in healthy aging, Alzheimer's disease, and Parkinson's disease

**DOI:** 10.1002/hbm.26171

**Published:** 2022-12-14

**Authors:** Sevilay Ayyildiz, Halil Aziz Velioglu, Behcet Ayyildiz, Bernis Sutcubasi, Lutfu Hanoglu, Zubeyir Bayraktaroglu, Suleyman Yildirim, Alper Atasever, Burak Yulug

**Affiliations:** ^1^ Anatomy PhD Program, Graduate School of Health Sciences Kocaeli University Kocaeli Turkey; ^2^ Department of Anatomy, School of Medicine Istinye University Istanbul Turkey; ^3^ Science for Life Laboratory, Department of Women's and Children's Health Karolinska Institute Stockholm Sweden; ^4^ Functional Imaging and Cognitive‐Affective Neuroscience Lab (fINCAN) Health Sciences and Technology Research Institute (SABITA), Regenerative and Restorative Medicine Research Center (REMER), Istanbul Medipol University Istanbul Turkey; ^5^ Department of Psychology, Faculty of Arts and Sciences Acibadem University Istanbul Turkey; ^6^ Department of Neurology, School of Medicine Istanbul Medipol University Istanbul Turkey; ^7^ Istanbul Medipol University International School of Medicine, Department of Physiology Istanbul Turkey; ^8^ Department of Medical Microbiology International School of Medicine, Istanbul Medipol University Istanbul Turkey; ^9^ Istanbul Medipol University International School of Medicine, Department of Anatomy Istanbul Turkey; ^10^ Department of Neurology, School of Medicine Alanya Alaaddin Keykubat University Antalya Turkey

**Keywords:** Alzheimer's disease, claustrum, elderly people, functional connectivity, Parkinson's disease

## Abstract

The claustrum is a sheet‐like of telencephalic gray matter structure whose function is poorly understood. The claustrum is considered a multimodal computing network due to its reciprocal connections with almost all cortical areas as well as subcortical structures. Although the claustrum has been involved in several neurodegenerative diseases, specific changes in connections of the claustrum remain unclear in Alzheimer's disease (AD), and Parkinson's disease (PD). Resting‐state fMRI and T1‐weighted structural 3D images from healthy elderly (*n* = 15), AD (*n* = 16), and PD (*n* = 12) subjects were analyzed. Seed‐based FC analysis was performed using CONN FC toolbox and T1‐weighted images were analyzed with the Computational Anatomy Toolbox for voxel‐based morphometry analysis. While we observed a decreased FC between the left claustrum and sensorimotor cortex, auditory association cortex, and cortical regions associated with social cognition in PD compared with the healthy control group (HC), no significant difference was found in alterations in the FC of both claustrum comparing the HC and AD groups. In the AD group, high FC of claustrum with regions of sensorimotor cortex and cortical regions related to cognitive control, including cingulate gyrus, supramarginal gyrus, and insular cortex were demonstrated. In addition, the structural results show significantly decreased volume in bilateral claustrum in AD and PD compared with HC. There were no significant differences in the claustrum volumes between PD and AD groups so the FC may offer more precise findings in distinguishing changes for claustrum in AD and PD.

## Graphical abstract

The claustrum is functionally connected to regions associated with characterized networks such as salience, default mode, executive, sensorimotor, visual, and language in the HC.

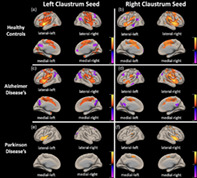

**Abbreviations**
AD: Alzheimer's disease
ART: artifact detection tools
CAT12: Computational Anatomy Toolbox
CSF: cerebrospinal fluid
FC: functional connectivity
FOC: frontal orbital cortex
GM: gray matter
HC: healthy control group
IFG: inferior frontal gyrus
ITG: inferior temporal gyri
LOC: lateral occipital cortex
MFG: middle frontal gyri
MMSE: Mini‐Mental State Examination
MNI: Montreal Neurological Institute
MTG: middle temporal gyri
PaCiG: paracingulate gyrus
PD: Parkinson's disease
ROI: region of interest
rs‐fMRI: resting‐state functional magnetic resonance imaging
SFG: superior middle frontal gyri
SMA: supplementary motor area
SMG: supramarginal gyrus
SPG: superior parietal gyrus
SPM12: Statistical Parametric Mapping 12
SRCC: Small Region Confound Correction
STG: superior temporal gyrus
TIV: total intracranial volume
WM: white matter

## 1 INTRODUCTION

The claustrum is a thin, irregular‐shaped and enigmatic subcortical structure located in the deep parts of both the cerebral hemispheres. It exhibits widespread, reciprocal connectivity with many cortical regions including motor, somatosensory, visual, auditory, limbic, and associative prefrontal cortices as well as subcortical structures. Although the volume of the claustrum is estimated about 0.2% of the total volume of the cerebral cortex (Kowiański et al., 1999; Moryś et al., 1996) it has the highest connectivity per unit volume among all the regions of the brain (Torgerson et al., 2015). Studies have suggested that the claustrum mediates a variety of functions, from motor to cognitive. However, direct functional assessments remain largely theoretical (Van Horn, 2019; White et al., 2020).

Several neuroanatomical studies point complex claustral connection network responsible for higher order cognitive functions (Wong et al., 2021). The hypothesized functions of the claustrum involve attention, cognition, consciousness (Crick & Koch, 2005), sensorimotor integration (Pathak & Fernandez‐Miranda, 2014), regulating exploratory behaviors (Smith & Alloway, 2014), saliency detection (Rodríguez‐Vidal et al., 2019; Smythies et al., 2014), and establishing new connections on memory and stress. The claustrum influences cortical activity as either an inhibitor or an excitator, especially in supporting optimal behavioral performance under cognitively demanding conditions (Jackson et al., 2018; White et al., 2020). Neuromodulation studies have proposed that, acting as a cortical conductor, the claustrum may fine‐tune inhibitory and/or excitatory tempos, depending on the current brain state (Crick & Koch, 2005; Wong et al., 2021).

Lesions of the claustrum can lead to depression, cognitive, and behavioral impairment because of its potential role in mediating the neural transmission (Smythies et al., 2014). Changes in the morphology and structural connectivity of the claustrum are seen in neurodegenerative diseases, such as Parkinson's disease (PD), Alzheimer's disease (AD), schizophrenia, depressive disorder, and hallucinations (Arrigo et al., 2019; Bruen et al., 2008; Cascella et al., 2011). Pathology of the claustrum has been linked to numerous potential cognitive dysfunctions and dementia in AD and PD (Arrigo et al., 2019; Morys et al., 1996). A decreased claustrum volume on the left side was positively correlated with the presence and severity of delusions in AD (Bruen et al., 2008). Additionally, the accumulation of amyloid plaques within the claustrum in AD is probably related to memory dysfunction and dementia (Morys et al., 1996; Ogomori et al., 1989). Cognitive decline in PD patients has been hypothetically attributed to decreased structural connectivity of the claustrum (Arrigo et al., 2019). The alpha‐Syn burden in the claustrum in PD cases with dementia was demonstrated that the claustrum may play a relevant role for presence of dementia in PD (Kalaitzakis et al., 2009). Reduction of dopamine and adrenaline levels in the claustrum in PD has been potentially linked to both motor and nonmotor symptoms observed in these patients (Sitte et al., 2017).

Neuroimaging studies, in both animal models and in humans, have proved extensive pattern of the claustrum connectivity with many cortical regions and subcortical structures (Arrigo et al., 2017; Krimmel et al., 2019; Smith et al., 2017; Torgerson et al., 2015). Resting‐state functional magnetic resonance imaging (rs‐fMRI) has revealed functional connectivity (FC) of the claustrum in healthy adult humans (Krimmel et al., 2019; Rodríguez‐Vidal et al., 2019). However, it is unknown whether any change occurs in the elderly or in some neurodegenerative diseases.

The purpose of this study to explore the resting‐state FC analyses with the time series of claustral ROI (Region of interest) to the rest of the brain in relation to elderly age, AD, and PD. We expected to find significantly altered FC in neurodegenerative diseases in comparison to healthy control group (HC).

## 2 METHODS

### 2.1 Subjects

The study included data from 16 AD subjects (9 women and 7 men average age: 70.31 ± 7.88) and 12 PD subjects (4 women and 8 men average age: 75.75 ± 10.52) without any neuropsychiatric symptoms. Furthermore, 15 healthy subjects (9 women and 6 men average age: 60.26 ± 9.37) without any history of neurological diseases were included as a control group and their Mini‐Mental State Examination (MMSE) score of 24 or higher (Güngen et al., 2002).

We retrospectively acquired data from the Neurology Departments of Istanbul Medipol University. Patients with AD who in Stage 1 or 2 according to the Clinical Dementia Scale and had no neurological or psychiatric disease other than AD were included. Individuals diagnosed with PD were in Stages 2–4 (Hoehn and Yahr Scale) and without serious mental or psychological disorder. All participants were right‐hand dominant. The studies received ethics approval of the Istanbul Medipol University (Ethical Report No: E‐10840098‐772.02‐6111).

### 2.2 Image acquisition

The structural and resting‐state functional MRI data acquired at Istanbul Medipol University using Philips Achieva 3 T (Philips Medical Systems, Best, Holland). Each subject T1‐weighted structural scans were 190 slices; (TR/TE: 8.1/3.7), FOV 256 × 256 × 190 mm (FHxAPxRL), voxel size was determined as 1 × 1 × 1 mm. The eyes‐open resting‐state fMRI scans recording lasted approximately 12 min and 300 volumes were recorded with following parameters: TR 2230 ms, TE 30 ms, FOV 240 × 240 × 140 mm (RLxAPxFH), voxel size 3 × 3 × 4 mm, flip angle 77°, and slices 35.

### 2.3 ROI definition

CONN toolbox does not comprise predefined seed for claustrum. Seed‐based FC analysis and volume analysis of claustrum were implemented with seeds manually defined by Krimmel et al. (Krimmel et al., 2019). Krimmel et al. individually drawn ROIs of left and right claustrum on the subject's normalized structural image and applied Small Region Confound Correction (SRCC) approach including isolating “flanking regions” of the brain neighboring the claustrum. (Krimmel et al., 2019).

### 2.4 rs‐fMRI processing

Seed‐based FC analysis was conducted using Conn toolbox version 18 (http://web.mit.edu/swg/software.htm) with SPM12 (Statistical Parametric Mapping 12) (http://www.fil.ion.ucl.ac.uk/spm/software/spm12/), in MATLAB 20b version (MathWorks, Sherborn, MA). The default preprocessing pipeline involved motion correction, co‐registration of a high‐resolution anatomic scan to the mean realigned functional image and spatial normalization to the 2 × 2 × 2mm Montreal Neurological Institute (MNI) standard brain template. The Artifact detection tools (ART) was performed to detect the outlier functional images (Mazaika et al., 2009). For optimal functional outlier detection, the conservative setting with 95th percentiles with linear motion parameters >0.5 mm and global‐signal *z* > 3 value threshold was adopted. The detected outlier scans and ages of the participants were defined as covariates to eliminate movement artifacts and ages related connectivity changes (Power et al., 2012). The structural images were segmented and separated into the gray matter (GM), white matter (WM), and cerebrospinal fluid (CSF) through tissue probability maps. After these preprocessing steps, the images were smoothed with an 8‐mm FWHM Gaussian kernel. In the denoising step, band‐pass filtering was applied with a frequency window of 0.008–0.09 Hz to reduce the influence of noise. The signals collected from WM and cerebrospinal fluid as an anatomical component‐based noise correction procedure (aCompCor), and the confounding factors derived from the resting state were subsequently regressed, as were the six motion parameters and their first‐order derivatives (Behzadi et al., 2007). In seed‐to‐voxel analysis, predefined two seeds by Krimmel et al. were added in the first‐level analysis. The ROIs used average time series as seed and then the analysis computed the correlation coefficients between these average time series and the BOLD time series at each voxel. For statistical analysis, these correlation coefficients were z‐transformed using the Fisher transformation. All ROI *z* values were compared across the three groups using ANOVA at the second level, with *p*_unc_ <0.001 at the voxel level and FWE adjusted *p*_FWE_ <0.05 at the cluster level. The Bonferroni‐corrected data were given after *p* values were corrected for multiple comparisons (*p*_FWE‐BC_: 0.025).

### 2.5 Structural processing

The high‐resolution 3D T1‐weighted images were processed with the Computational Anatomy Toolbox (CAT12: http://dbm.neuro.uni-jena.de/cat/), run through SPM12 in MATLAB (20b) for Voxel‐Based Morphometry (VBM) analysis (Gaser & Dahnke, 2016). The T1 images were affine registered, corrected for bias‐field inhomogeneities, and segmented into GM, WM, and CSF tissues according to the MNI template (the standard template included in SPM12) followed by the unified segmentation. Afterward, all of the tissue segments were modulated normalized to the standard MNI template (the standard included in SPM12). Besides, total intracranial volume (TIV) was estimated to correct for different brain sizes and volumes as well. GM images were smoothed with an 8‐mm FWHM Gaussian kernel via the standard SPM module “Smooth” to increase the signal‐to‐noise ratio. After the preprocessing step, a quality check was performed to assess the homogeneity of the GM tissues.

Group differences in GM volumes were compared by one‐way ANOVAs. As some researchers reported that the age and TIV of subjects have an impact on the VBM results, we used the subject's age and TIVs in the design matrix as covariates (Peelle et al., 2012). To address multiple comparisons, the results are reported after corrected as voxel‐level threshold: uncorrected *p* < .001, cluster level threshold: family‐wise error (FWE) *p* < .05.

## 3 RESULTS

There were significant differences between the groups in terms of age and MMSE score using ANOVA and post hoc (Bonferroni) tests and the 𝑝 < .05 was considered statistically significant (Table 1). Age and MMSE scores were included as a covariate in the FC analysis to control for their covariates by group interactions (one‐way ANCOVA). No age‐related differences were found for claustrum FC in groups. Besides, significant differences were observed between HC and PD, as well as AD and PD groups. Between‐group differences in FC of bilateral claustrum related to MMSE score are shown in Figure 2.

**TABLE 1:** Demographic data were given of ADs, PDs, and HC groups

	HC (*n* = 15)	ADs (*n* = 16)	PDs (*n* = 12)	*p*‐value (HC‐ADs)	*p*‐value (HC‐PDs)
Age	60.26 ± 9.37	70.31 ± 7.88	75.75 ± 10.52	.012	.000
Sex (F/M)	9/6	9/7	4/8	NS	NS
MMSE score	27.6 ± 1.80	18.09 ± 4.74	19.08 ± 3.98	.000	.000

*Note*: The mean difference is significant at the 0.017 level.

Abbreviation: NS, not significant.

### 3.1 Claustrum volume

The mean volumes and mean *p* values for group differences of right and left claustrum are included in Table 2. We identified the decreased GM volume in bilateral claustrum in AD and PD compared with HC. We obtained that the right claustrum had a larger volume than the left one in all the participants, but the difference was not significant. Differences in the claustrum volumes between the HC and AD as well as between HC and PD were statistically significant. There were no significant differences in the claustrum volumes between PD and AD groups.

**TABLE 2:** Volume (mm^3^) of the claustrum and mean difference between groups

				Mean differences *p*‐value	
	Control	Alzheimer's disease (ADs)	Parkinson's disease (PDs)	Control	PD
				AD PD	AD
Left Claustrum	447.173	367.907	392.275	.000 .002	NS
Right Claustrum	464.407	368.080	397.517	.000 .002	NS

*Note*: *p*‐value assesses the difference between groups by one‐way ANOVA with *post hoc* test for volume (*p* < .05).

Abbreviation: NS, not significant.

### 3.2 Analysis of resting‐state claustrum connectivity

**FIGURE 1:**
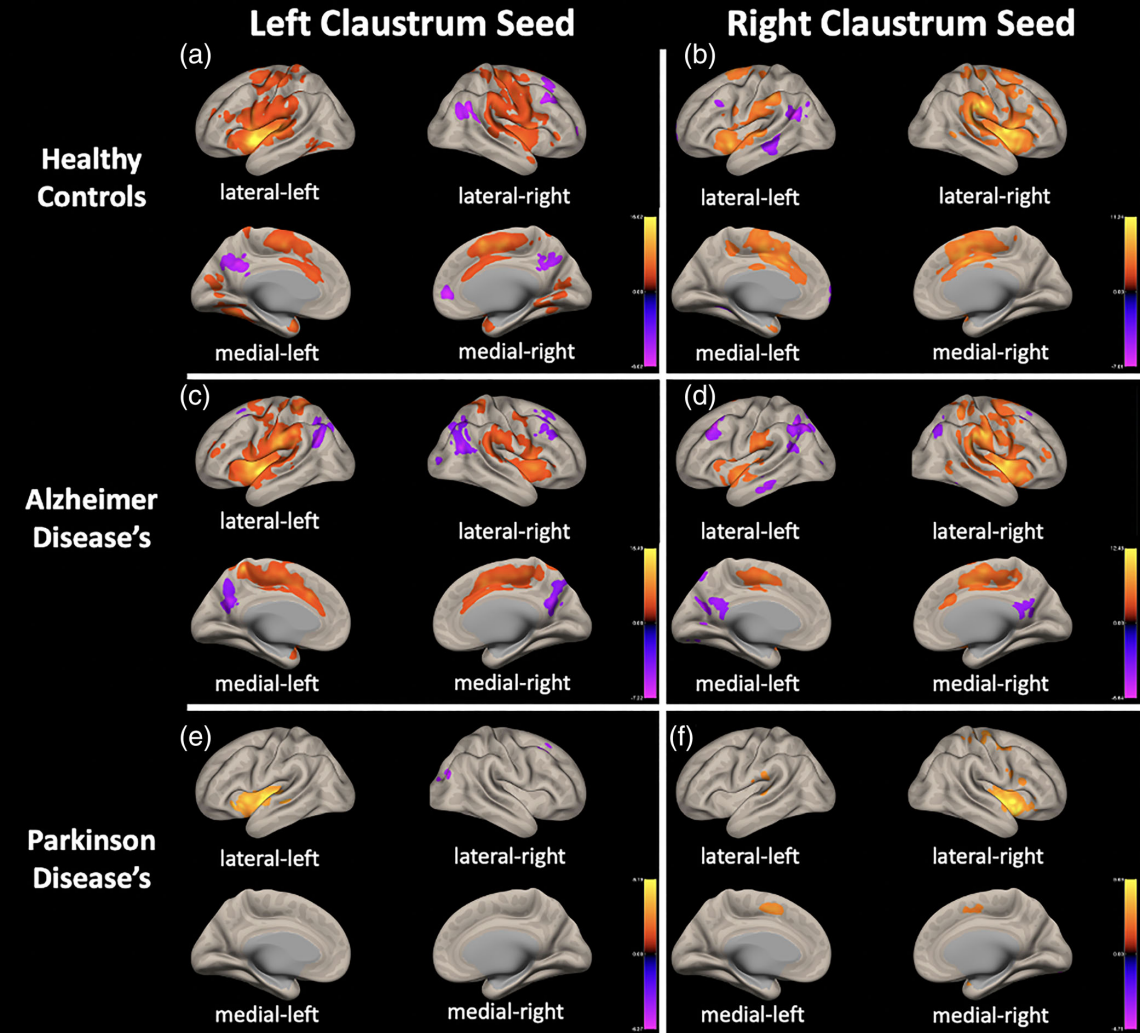
Seed‐to‐voxel functional connectivity with bilateral claustrum showing extensive connectivity to cortical and subcortical regions (*p* < .025, *p*‐FEW corrected). The within‐group connectivity patterns to the left claustrum seed are shown for healthy controls in (a), Alzheimer's Disease in (c), and Parkinson's disease in (e), and to the right seed in (b), (d), and (f)

We characterized the whole‐brain connectivity of the claustrum in HC, AD, and PD with seed‐based correlational analysis. The claustrum was found to have extensive FC with many cortical and subcortical regions in healthy controls. We found FC of the left claustrum with sensorimotor cortex (involving precentral and postcentral gyrus), supplementary motor area (SMA), cingulate cortex, insular cortex, superior (SFG) and middle frontal gyri (MFG), frontal orbital cortex (FOC), superior temporal gyrus (STG), paracingulate gyrus (PaCiG), superior parietal gyrus (SPG), parahippocampal gyrus (anterior division), lateral occipital cortex (LOC), opercular cortex, cuneus, precuneus, inferior frontal gyrus (IFG) (pars opecularis‐pars triangularis), fusiform gyrus, lingual gyrus, thalamus, amygdala, and cerebellum (crus2), as well as the middle (MTG) and inferior temporal gyri (ITG), putamen, pallidum, accumbens, caudate nucleus ipsilaterally, supramarginal gyrus (SMG), and angular cortex contralaterally. This analysis identified right claustral FC with the insular cortex, cingulate cortex, SFG, SMA, SMG, and putamen bilaterally. The FC between right claustrum and sensorimotor cortex, IFG (pars opercularis), opercular cortex, PaCiG, and SPG was ipsilateral; and MTG, lingual gyrus, angular gyrus, LOC, MFG, hippocampus, and cerebellum were contralateral. Although in AD the regions displaying FC with claustrum were largely similar with HC, decreased claustral connectivity in PD was observed in comparison to other groups (Figure 1).

In AD, we identified FC of the right and left claustrum (*p* < .05 p‐FWE corrected) with sensorimotor cortex, SMA, insular cortex, frontal cortex, cingulate cortex, SMG, angular gyrus, PaCiG, opercular cortex, SPG, STG, precuneus cortex, LOC (superior division), putamen, thalamus, pallidum, and cerebellum (crus1 and 2) bilaterally, amygdala ipsilaterally. The right claustrum has projection right claustrum MTG, ITG, and right LOC (inferior division), as well as there is connectivity between left claustrum and left caudate nucleus (Figure 1).

While the FC were similar across both side claustrum in PD, right claustrum overall had more extensive resting‐state FC. We found FC between the left claustrum and insular cortex, putamen, pallidum, STG, opercular cortex, FOC ipsilaterally, and SFG, MFG, and LOC (superior division) contralaterally. Right claustrum had an FC with insular cortex, pallidum, precentral gyrus, amygdala, IFG, FOC on the right side, and STG, PaCiG on the left side, and putamen, opercular cortex, SMA, and postcentral gyrus on both sides (Figure 1).

### 3.3 Group differences in connectivity

**FIGURE 2:**
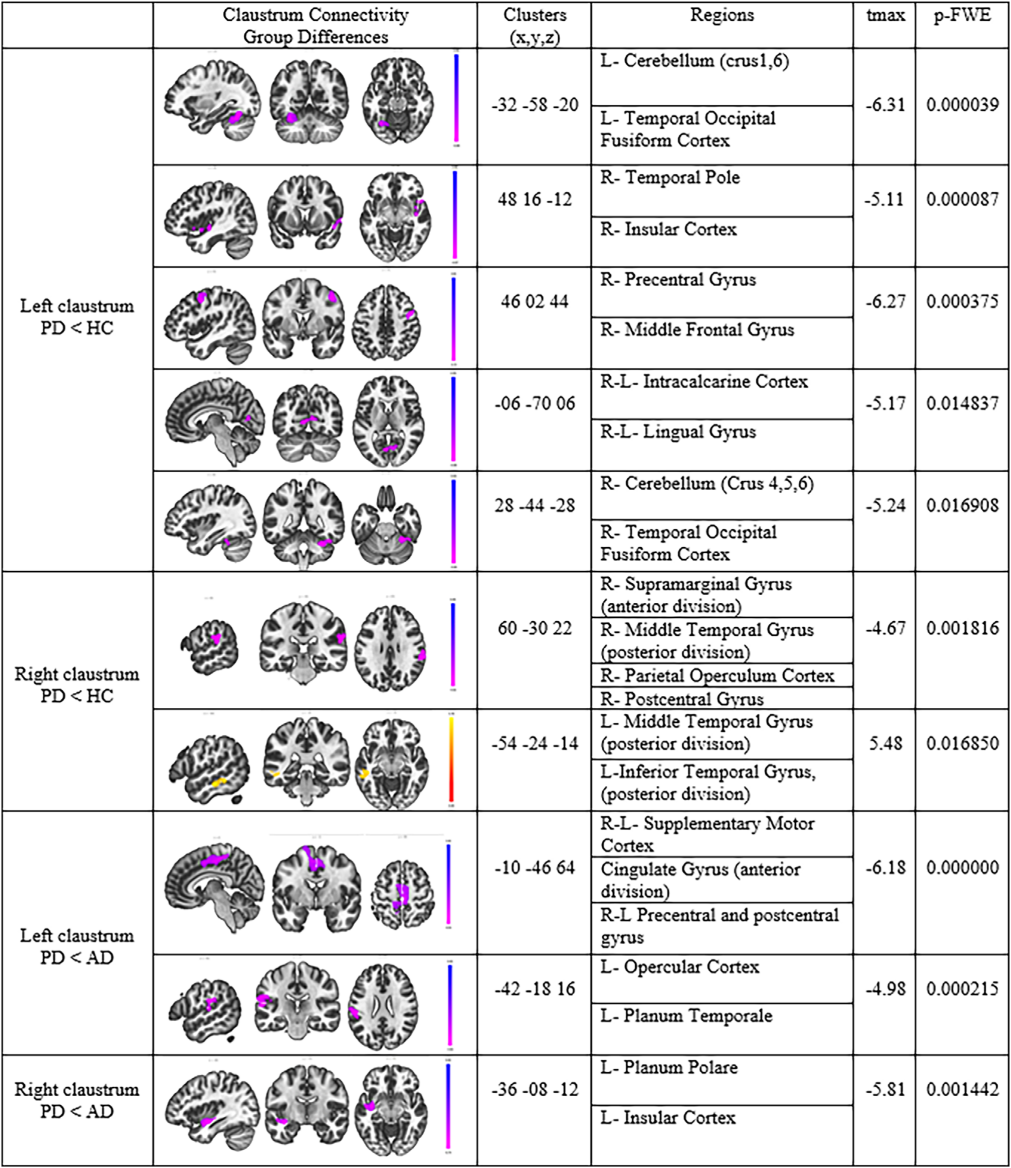
Clusters of voxels with significant intergroup functional connectivity differences. *p*‐value assesses the difference between groups with MMSE score covariate by one‐way ANCOVA (*p* < .001 voxel and *p*‐FWE <0.025 at cluster level)

**FIGURE 3:**
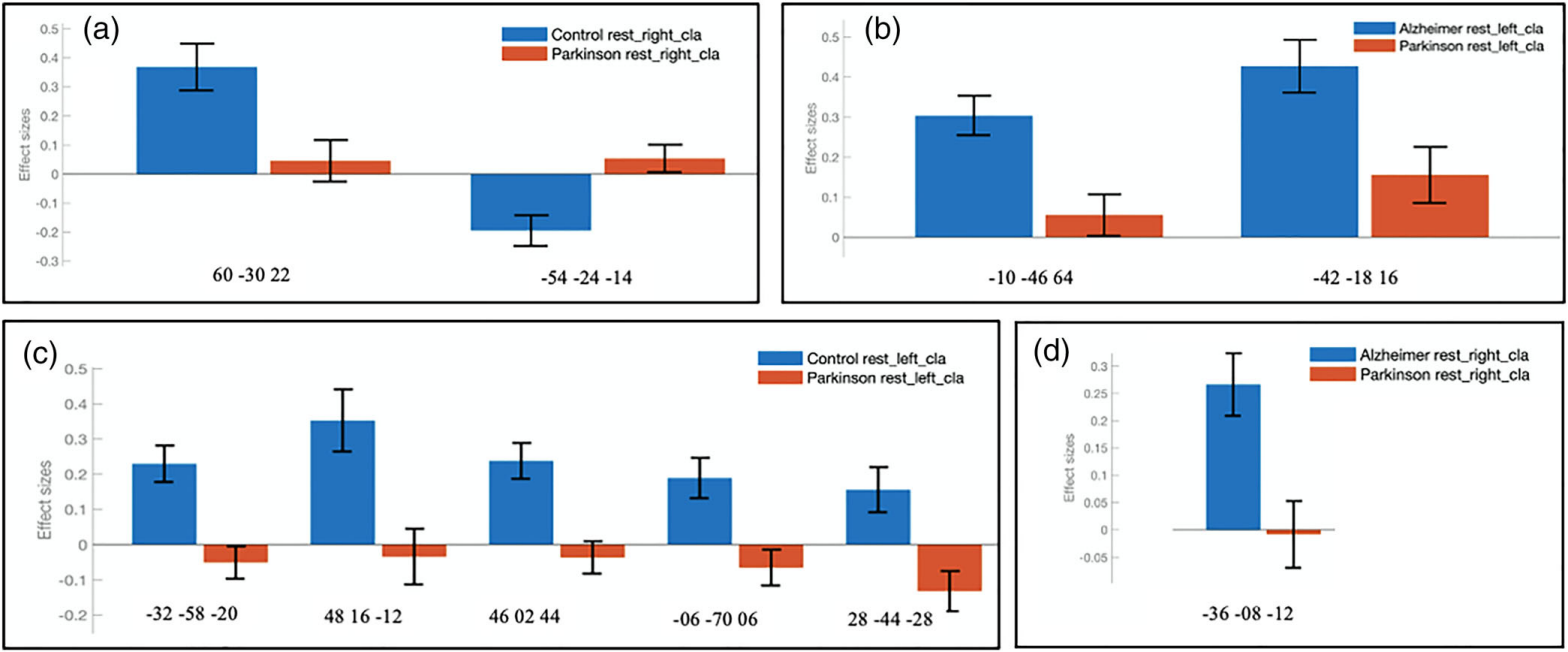
Effect size of claustrum functional connectivity difference between healthy control, Alzheimer's disease, and Parkinson's disease groups. The regions to which the MNI coordinates correspond are described in Figure 2. Group differences are shown for the right claustrum seed for healthy control (HC) > Parkinson's disease (PD) in (a), for the left claustrum seed for Alzheimer's disease (AD) > (PD) in (b), for the left claustrum seed for (HC) > (PD) in (c), for the right claustrum seed for (AD) > (PD) in (d)

The voxel‐wise connectivity values among HC, AD, and PD groups resulted in significant effects in two predefined ROIs (Figure 2, Figure 3). While we found a widespread decrease in FC in PD compared with HC, no significant difference was found in the FC for the claustrum in both hemispheres comparing the HC and AD groups.

In comparison to HC and PD manifested decreased FC between claustrum and sensorimotor cortex, insular cortex, fusiform cortex, temporal pole, lingual gyrus, SMG, MTG, ITG, and cerebellum. Comparing the connectivity of the claustrum between AD and PD exhibited decreased FC of the claustrum with regions of sensorimotor cortex, insular cortex, cingulate gyrus, and opercular cortex in PD.

## 4 DISCUSSION

This study analyzed FC of right and left claustrum in a group of AD, PD, and healthy elderly individuals (Figure 1). The results showed that the claustrum is strongly functionally connected to sensorimotor, prefrontal, cingulate, and insular cortices in all groups. Extensive connectivity of both claustrum to numerous cortical and subcortical regions was observed in HC and AD group. However, the FC in the PD claustrum was significantly lower than in the other groups. Although claustrum connectivity values were similar between the healthy control and AD groups, claustrum connectivity in PD was significantly lower in comparison to the other groups (Figures 2 and 3).

Our findings reveal that the claustrum is functionally connected to regions associated with characterized networks such as salience, default mode, executive, sensorimotor, visual, and language in the healthy elder group. The FC of the claustrum patterns have been reported to involve executive and sensorimotor cortical regions including frontal, temporal, cingulate, and occipital cortices as well as thalamus, accumbens, hippocampus, caudate nucleus, amygdala, and putamen in young adults (Krimmel et al., 2019; Rodríguez‐Vidal et al., 2019). Although these data are in keeping with our results, we observed FC between the claustrum and cuneus, pallidum, lingual gyrus, PaCiG, SPG, and cerebellum in older adults, in contrast to previous studies of young adults. To our knowledge, the present research is the first to explore the FC of the claustrum in healthy elderly individuals. In addition, volumes of 813.6 and 804.0 mm^3^ have been reported for the right and left claustrum, respectively (Milardi et al., 2015). We found dramatic differences in the rate of decline of claustrum volume in healthy older adult. Claustrum shrinkage has also been described in neuropsychiatric disorders (Davis, 2008). However, claustral size correlates highly with whole brain size (Kowiański et al., 1999). Further studies are now needed to clarify the relationship between reduced volume of the claustrum and the density of connections and cognitive involvement.

The claustrum is associated with numerous high‐level brain functions. Changes in claustrum therefore may result in cognitive and behavioral symptoms in AD and PD, common neurodegenerative disorders (Bruen et al., 2008; Kalaitzakis et al., 2009). The claustrum receives large numbers of inputs from numerous brain regions, including the parietal and medial temporal regions, and projects to the insula and frontal pole (Burman et al., 2011; Torgerson et al., 2015). Claustrum dysfunction has thus been linked to disruption in episodic memory formation (Schwindt & Black, 2009), attentional management (Atlan et al., 2018), consciousness (Koubeissi et al., 2014), cognitive functions (Krimmel et al., 2019), spatial navigation (Jankowski & O'Mara, 2015), and mechanisms of information processing across sensory modalities (Nikolenko et al., 2021), sexual functions (Redouté et al., 2000), and esthetic judgment (Ishizu & Zeki, 2013).

Older age does not cause AD, although rising age is believed to increase the risk of the disease since it is related to brain atrophy (Raji et al., 2009). The studies evaluating regional morphologic brain changes in normal aging and AD have not reported altered GM density in the claustrum (Ohnishi et al., 2001; Raji et al., 2009). However, we observed significant volume decrease in AD compared with HC. A decreased volume of the claustrum is also associated with the presence of delirium, progression of dementia, and cognitive decline (Nikolenko et al., 2021). Abnormalities of the claustrum and insula have also been linked to the presence of positive symptoms in both neurodegenerative and psychiatric conditions (Venneri & Shanks, 2014). Low GM density values have been observed in the left claustrum in AD with behavioral and neuropsychiatric symptoms and high delusion scores in these patients were associated with claustrum atrophy (Bruen et al., 2008). An fMRI study involving connectivity and memory described the claustrum as one of the areas of common deactivations in AD patients and healthy older adults. As a result of this connectivity problem, claustrum may have an effect to fail the associates learning paradigm, in the encoding and retrieval stages of AD patients and older adults (Gould et al., 2006). No resting‐state change in FC of the claustrum was observed between the AD patients and healthy controls in the present study.

Neuropathological and neuroimaging studies have implicated pathology of the claustrum in progressive dementia in PD (Arrigo et al., 2019; Sitte et al., 2017). The severe loss of claustrum dopamine and noradrenaline can lead to deterioration of cortico‐claustral‐cortical circuitry, thus producing motor and nonmotor symptoms (Sitte et al., 2017). Arrigo et al. suggested that decreased claustral structural network might be linked to cognitive and default mode network dysfunction as well as memory and executive task alterations in PD (Arrigo et al., 2019). Contrary to expectations, this study found that significant decreased FC of claustrum with sensorimotor cortical regions including postcentral gyrus, precentral gyrus, and SMA in PD compared with HC and AD. Previous studies have suggested that the claustrum synchronizes different sensory and motor modalities to give rise to conscious percepts (Crick & Koch, 2005) and coordinates sensorimotor information (Smith et al., 2012). Studies have also suggested that deficits in array of sensory processing and integration of proprioceptive and other sensory inputs may lead to degradation of motor function in PD (Konczak et al., 2009; Taghizadeh et al., 2018). Kavounoudias et al. observed activation of the claustrum during proprio‐tactile stimulation performed to generate kinesthetic illusion perception (Kavounoudias et al., 2008). Hence, it could conceivably be hypothesized that decreased FC between the claustrum and sensorimotor cortical regions may impact adversely on the processing of kinesthetic information in PD.

Deficiency in emotional and cognitive social processes has been observed in PD; these include recognition of emotion and facial expression, empathy, understanding complex social interactions, and executive function abilities (Maresca et al., 2020; Narme et al., 2013). It has also been postulated that the claustrum performs a mediating role in cognition‐related processes (Crick & Koch, 2005; Nikolenko et al., 2021). In the current study, there was a significant decrease in FC between claustrum and cortical regions associated with social cognition, including insular cortex and temporal pole in the right hemisphere in PD compared with HC.

Although no significant differences were determined in claustrum volume between AD and PD, a significant difference was observed in claustral FC. Surprisingly, we observed decreased FC of claustrum with the sensorimotor, anterior cingulate, insular, and opercular cortex in PD compared with AD. Accordingly, these findings suggest that alteration in FC of claustrum may be considered as a more distinguishable factor than volumetric change.

Overall, the regions displaying FC with claustrum in HC have supported the previous hypotheses concerning the function of the claustrum. Although we observed significant differences between PD and other groups, surprisingly there were no significant differences in AD. These differences may be explained by the dissimilar neuropathology and claustral chemical neurotransmission in AD and PD. According to Sitte et al.'s hypothesis, dopaminergic projections extend to the claustrum from the substantia nigra compacta, with the claustrum playing a potential role as a bypass station to facilitate the transmission of integrated information to the cortex via direct connections. Furthermore, claustral dopamine deficit disrupts the function of the claustrum in PD (Sitte et al., 2017). The insignificant claustral FC differences between AD and HC may be attributable to brain atrophy mechanisms in aging and AD. We hypothesize that claustrum mediates sensorimotor information and social cognition process. However, several questions remain unanswered. Further studies with greater focus on the effects of neuromodulator control of the claustrum in neurodegenerative diseases are therefore recommended.

The present study is the first to have demonstrated and compared FC of claustrum in healthy aging, AD, and PD. We believe this study makes a noteworthy contribution to research on FC of claustrum in elderly adults, AD, and PD.

## AUTHOR CONTRIBUTIONS

Sevilay Ayyildiz was involved in conceptualization (equal), data curation (lead), formal analysis (equal), validation (equal), and writing—original draft (equal). Halil Aziz Velioglu was involved in conceptualization (equal), data curation (lead), formal analysis (equal), validation (equal), and writing—original draft (equal). Behcet Ayyildiz was involved in conceptualization (supporting); formal analysis (equal), and software (equal). Bernis Sutcubasi was involved in conceptualization (supporting), methodology (equal), formal analysis (equal), and visualization (equal). Lutfu Hanoglu was involved in conceptualization (supporting), investigation (equal), and validation (equal). Zubeyir Bayraktaroglu was involved in conceptualization (supporting), methodology (supporting), and investigation (equal). Suleyman Yildirim was involved in investigation (equal), methodology (supporting), and resources (equal). Burak Yulug was involved in conceptualization (supporting), and writing—review and editing (supporting). Alper Atasever was involved in conceptualization (supporting) and writing—review and editing (supporting).

## CONFLICT OF INTEREST

No conflict of interest to report.

## Data Availability

Data available on request from the authors
